# Specific biomarker mining and rapid detection of *Burkholderia cepacia* complex by recombinase polymerase amplification

**DOI:** 10.3389/fmicb.2023.1270760

**Published:** 2023-09-14

**Authors:** Yiling Fan, Shujuan Wang, Minghui Song, Liangliang Zhou, Chengzhi Liu, Yan Yang, Shuijing Yu, Meicheng Yang

**Affiliations:** ^1^China State Institute of Pharmaceutical Industry, Shanghai, China; ^2^National Medical Products Administration Key Laboratory for Testing Technology of Pharmaceutical Microbiology, Shanghai Quality Inspection and Testing Center for Innovative Biological Products, Shanghai Institute for Food and Drug Control, Shanghai, China; ^3^College of Resource and Environmental Engineering, Jiangxi University of Science and Technology, Ganzhou, Jiangxi, China; ^4^Department of Infectious Diseases, Sir Run Run Shaw Hospital, College of Medicine, Zhejiang University, Hangzhou, China; ^5^Hangzhou Digital-Micro Biotech Co., Ltd., Hangzhou, China; ^6^Shanghai Food and Drug Packaging Material Control Center, Shanghai, China

**Keywords:** *Burkholderia cepacia* complex, molecular marker, mining, *secY* gene, recombinase polymerase amplification, rapid detection

## Abstract

**Objective:**

To mine specific proteins and their protein-coding genes as suitable molecular biomarkers for the *Burkholderia cepacia* Complex (BCC) bacteria detection based on mega analysis of microbial proteomic and genomic data comparisons and to develop a real-time recombinase polymerase amplification (rt-RPA) assay for rapid isothermal screening for pharmaceutical and personal care products.

**Methods:**

We constructed an automatic screening framework based on Python to compare the microbial proteomes of 78 BCC strains and 263 non-BCC strains to identify BCC-specific protein sequences. In addition, the specific protein-coding gene and its core DNA sequence were validated *in silico* with a self-built genome database containing 158 thousand bacteria. The appropriate methodology for BCC detection using rt-RPA was evaluated by 58 strains in pure culture and 33 batches of artificially contaminated pharmaceutical and personal care products.

**Results:**

We identified the protein SecY and its protein-coding gene *secY* through the automatic comparison framework. The virtual evaluation of the conserved region of *the secY* gene showed more than 99.8% specificity from the genome database, and it can distinguish all known BCC species from other bacteria by phylogenetic analysis. Furthermore, the detection limit of the rt-RPA assay targeting the *secY* gene was 5.6 × 10^2^ CFU of BCC bacteria in pure culture or 1.2 pg of BCC bacteria genomic DNA within 30 min. It was validated to detect <1 CFU/portion of BCC bacteria from artificially contaminated samples after a pre-enrichment process. The relative trueness and sensitivity of the rt-RPA assay were 100% in practice compared to the reference methods.

**Conclusion:**

The automatic comparison framework for molecular biomarker mining is straightforward, universal, applicable, and efficient. Based on recognizing the BCC-specific protein SecY and its gene, we successfully established the rt-RPA assay for rapid detection in pharmaceutical and personal care products.

## 1. Introduction

The *Burkholderia cepacia Complex* (BCC) bacteria are a group of incredibly diverse but closely related species of Gram-negative, aerobic, non-fermentative bacteria that belong to the *β* subclass of the phylum *Proteobacteria* (Lessie et al., 1996; Mahenthiralingam et al., 2008; Tavares et al., 2020). BCC bacteria are spread worldwide and are proven to proliferate or survive in oligotrophic environments for an extended period (Mahenthiralingam et al., 2000; Ahn et al., 2014, 2019). They are also considered significant opportunistic human pathogens that produce a variety of potential virulence factors (Mahenthiralingam et al., 2005) and could cause devastating infections in patients with bacteremia, septic arthritis, bacterial peritonitis, and cystic fibrosis that lead to high mortality in infected and immunocompromised patients (Mahenthiralingam et al., 2008; Gautam et al., 2011; Datta et al., 2020; Wang and Cissé, 2020). BCC bacteria that could exhibit multiple antibiotic resistances are also essential pathogens of hospital-acquired infections (Decicco et al., 2010; Sousa et al., 2011; Sfeir, 2018).

Due to their nature and broad distribution, BCC bacteria have been proposed as objectionable organisms in warning letters, safety alerts, or recalls by the United States Food and Drug Administration. Global recalls related to BCC bacteria contamination were found frequently in water-based products, especially in pharmaceutical products, personal care products, and disinfectants (Kuhn et al., 1982; Balkhy et al., 2005; De Volder and Teves, 2020; Tavares et al., 2020; Zou et al., 2020; Du et al., 2021). BCC isolates, especially *B. cepacia*, were identified in 22% of those recalls as the leading cause of microorganisms in non-sterile product contamination from 2004 to 2011 (Jimenez, 2007; Sutton and Jimenez, 2012). To date, 24 species have been identified and classified as the *Burkholderia cepacia Complex* (Coenye et al., 2001c; Sfeir, 2018), of which nine genomovars (I–IX) are commonly isolated from water-based products or clinical samples. They are known as *B. cepacia* (I)*, B. multivorans* (II), *B. cenocepacia* (III)*, B. stabilis* (IV), *B. vietnamiensis* (V), *B. dolosa* (VI)*, B. ambifaria* (VII), *B. anthina* (VIII) and *B. pyrrocinia* (IX; Vandamme et al., 1997; Balandreau, 2001; Coenye et al., 2001c; Vanlaere et al., 2008; Vandamme and Dawyndt, 2011; Depoorter et al., 2020).

In 2020, the United States Pharmacopoeia (USP 43) published the official general chapter <60> to describe the test in non-sterile pharmaceutical products by a culture-based method. Indeed, this new chapter has gained a broad interest in recent years and is driving progress in quality risk management in pharmaceutical industries. However, this culture-based method is time-consuming and laborious, costing more than 4 to 7 days for a laboratory to acquire positive or negative results (Steinmetz et al., 1999; Amornchai et al., 2007). Therefore, a rapid, sensitive, accurate, and affordable detection technology for BCC bacteria is urgently needed in scientific research, survey, industries, and regulatory authorities.

Rapid molecular methods targeting specific genes provide tremendous advantages over conventional approaches (Attia et al., 2016; Sfeir, 2018). Polymerase chain reaction (PCR) is considered one of the most valuable techniques for rapid detection in decades. However, it takes ineffective time for dramatic temperature changes between each amplification cycle. Several isothermal amplification methods for nucleic acid have been introduced in forensics, animal and human pathogens over the last 10 years (Soroka and Wasowicz, 2021), such as loop-mediated isothermal amplification (LAMP), nucleic acid sequence-based amplification (NASBA), helicase-dependent amplification (HDA) and rolling circle amplification (RCA; Asiello and Baeumner, 2011; Zanoli and Spoto, 2013). In particular, the recombinase polymerase amplification (RPA) approach is carried out at constant room temperature between 38 and 42°C, which allows no need for a heating and cooling process. Therefore, it offers a simple reaction system with high sensitivity and selectivity compared to other methods (Piepenburg et al., 2006; Li et al., 2018; Lobato and O'Sullivan, 2018). These characteristics offer a tremendous molecular tool for daily primary screening (Craw and Balachandran, 2012; Zanoli and Spoto, 2013).

The *16S rDNA* gene*, recA* gene*, fur* gene, and *hisA* gene are commonly used gene targets for DNA typing and identification of *Burkholderia* species due to their genetic polymorphism (Campbell et al., 1995; Karlin et al., 1995; Eisen, 1996; Vandamme et al., 1996; Vermis et al., 2002; Lynch and Dennis, 2008; Papaleo et al., 2010; Devanga Ragupathi and Veeraraghavan, 2019). With the continuous discovery of novel species, unknown BCC bacteria tested by assays based on non-specific genes may cause misleading results (LiPuma et al., 1999). It is unlikely to cover all potential BCC strains based on highly polymorphic genes with one screening method. The lack of specific genes with fewer variants has significantly influenced the development of rapid detection for BCC bacteria in the pharmaceutical and cosmetics industries. With the robust growth of whole genome sequencing and bioinformatics analysis, the resources of microbial proteomes and genomes are available for high throughput comparative approaches to mine molecular biomarkers in a considerably short time, which are more economical and convenient for practical uses (Yu et al., 2011; Chen et al., 2019; Zhang et al., 2020; Srijuntongsiri et al., 2022). Therefore, it is necessary and possible to find a suitable molecular target for the complicated BCC bacteria using new mining strategies to avoid most of the deficiencies of false negative and false positive results (Yu et al., 2011).

In this study, we aimed to mine and discover a BCC-specific molecular target through an automatic high-throughput proteomic comparison and screening framework. After *in silico* evaluation with our database, the core conserved sequence of the protein-coding gene was introduced as a suitable biomarker of BCC bacteria for rapid real-time PRA assay. Furthermore, the screening testing method was developed and validated using artificially contaminated non-sterile pharmaceutical and personal care products.

## 2. Materials and methods

### 2.1. Bacterial strains and DNA extraction

Our study used 58 strains containing 34 BCC strains and 24 non-BCC strains for method validation (Table 1). Twenty-nine reference strains were purchased from the American Type Culture Collection (ATCC), the China Center of Industrial Culture Collection (CICC), the Guangdong Institute of Microbiology (GIM), and the China General Microbiological Culture Collection Center (CGMCC), respectively. The other 29 isolated strains were collected and separated from clinical patients, pharmaceutical products, and personal care products by the Shanghai Institute for Food and Drug Control. All bacterial strains were enriched using Soybean–casein digest agar (SCDA, Merck, USA) overnight at 36°C ± 1°C, and identified by Autoflex max MALDI-TOF mass (Bruker, USA) or VITEC 2 Compact (Biomerieux, France). Total genomic DNA from 1 ml of each fresh culture was extracted using the DNeasy Blood and Tissue Kit (QIAGEN, USA) directed by the manufacturer’s manual, then washed the template DNA with 100 μl of Tris-EDTA buffer solution and stored at −20°C for use. The quantity and purity of the bacterial genomic DNA were assessed using BioPhotometer Plus (Eppendorf, USA).

**Table 1:** List of bacterial strains tested in this study.

Bacterial strains	Genomovars	Source^a^	Quantities	RPA Results
*B. cepacia*	I	CICC 10857	1	+^c^
		CICC 20700	1	+
		Patient^b^	10	+
		Drug^b^	6	+
		Cosmetic^b^	3	+
*B. multivorans*	II	Drug^b^	1	+
*B. cenocepacia*	III	CICC 20699	1	+
		Cosmetic^b^	1	+
		Drug^b^	3	+
*B. stabilis*	IV	ATCC BAA-67	1	+
*B. vietnamiensis*	V	Drug^b^	1	+
		Patient^b^	1	+
*B. dolosa*	VI	ATCC BAA-246	1	+
*B. ambifaria*	VII	CGMCC 1.10511	1	+
*B. anthina*	VIII	Patient^b^	1	+
*B. pvrrocinia*	IX	Patient^b^	1	+
*B. tropica*	/^d^	CICC 10405	1	−
*B. unamae*	/	CICC 10406	1	−
*B. nodosa*	/	CICC 10407	1	−
*B. metalliresistens*	/	CICC 10561	1	−
*Pseudomonas putida*	/	CICC 10368	1	−
*Pseudomonas stutzeri*	/	CICC 10402	1	−
*Pseudomonas hunanensis*	/	CICC 10558	1	−
*Pseudomonas chengduensis*	/	CICC 20543	1	−
*Pseudomonas chlororaphis*	/	CICC 20676	1	−
*Pseudomonas alcaligenes*	/	CICC 20698	1	−
*Pseudomonas nitroreducens*	/	CICC 20703	1	−
*Pseudomonas saponiphila*	/	CICC 21462	1	−
*Pseudomonas straminea*	/	CICC 21628	1	−
*Pseudomonas mendocina*	/	CICC 21629	1	−
*Pseudomona aeruginosa*	/	CICC 21636	1	−
*Pseudomonas rhodesiae*	/	CICC 21961	1	−
*Pseudomonas lurida*	/	CICC 22029	1	−
*Pseudomonas plecoglossicida*	/	CICC 22994	1	−
*Rhodopseudomonas palustris*	/	CICC 23812	1	−
*Pseudomonas baetica*	/	CICC 23894	1	−
*Pseudomonas japonica*	/	CICC 23895	1	−
*Pseudomonas aeruginosa*	/	CICC 10351	1	−
*Brevundimonas diminuta*	/	Drug^b^	1	−
*Pseudomona fluorescens*	/	GIM 1.776	1	−

a ATCC: the American Type Culture Collection; CICC: the China Center of Industrial Culture Collection; GIM: the Guangdong Institute of Microbiology; CGMCC: the China General Microbiological Culture Collection Center.

b Samples collected by the Shanghai Institute for Food and Drug Control collected and separated isolated strains.

c +: a positive result of real-time RPA assay targeting the *secY* gene; −: a negative result of real-time RPA assay targeting the *secY* gene.

d /: Not applicable.

### 2.2. BCC biomarker mining

**Figure 1:**
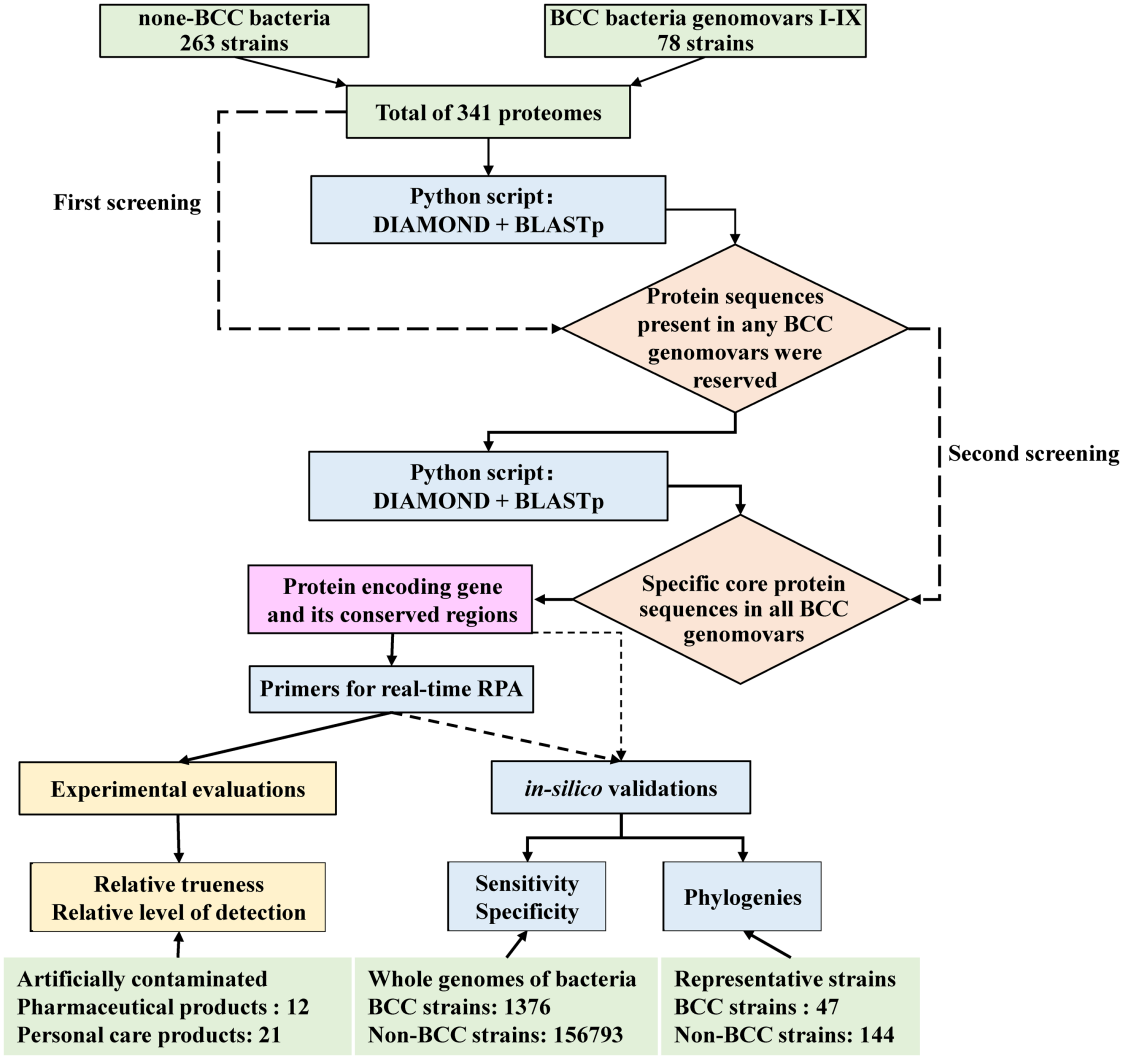
The automatic screening and evaluation framework for BCC-specific biomarker mining.

The proteomes of 341 bacterial strains for biomarker mining, including 78 BCC strains and 263 non-BCC strains, were downloaded from GenBank of the National Center for Biotechnology Information.^1^ The BCC biomarker screening framework from the proteomes database is shown in Figure 1. The protocol contained a secondary strategy to screen BCC-specific protein sequences. The first screening step is to automatically align the protein sequences from 341 bacterial strains using the pairwise comparison function of DIAMOND software (version 0.9.26; Buchfink et al., 2015) by a Python script. Protein sequences with low matching rates were excluded. The model parameter of the DIAMOND software was set as follows: E-value = 1 × e^−10^ and Identify = 98. Protein sequences matching any of the nine BCC genomovars in the pool were reserved. The second screening step is to automatically screen out core protein sequences that appeared in all BCC genomovars using the same function of DIAMOND software (E-value = 1 × e^−10^, and Identify was set as 90, 92, 94, and 98, respectively). The bioinformatic pipeline of biomarker mining was published on GitHub.^2^ The specificity of the remaining sequence from the second step was initially confirmed using BLASTp (version 2.13.0) on NCBI website databases.^3^ Then, the potential protein and its protein-coding gene sequence were extracted as biomarkers for the following primer design and *in silico* validation procedures.

### 2.3. Primer design for real-time RPA assay

One hundred and one sequences of the *secY* genes from different BCC stains were downloaded from the GenBank database for primer design. The software MEGA X was used to locate the relatively conserved regions by aligning the gene sequences, and the degenerate bases were plotted according to the alignment information. The primers and probes were designed using Primer Premier 5.0 according to the principles of TwistDx Limited (Table 2). The fluorescence DNA isothermal rapid amplification kit (Amp Future Biotechnology Co., LTD., China) was introduced for the real-time RPA assay. In detail, adding 29.5 μl of buffer A to pre-dried reaction powder from the kit to prepare reaction buffer A. Real-time RPA was carried out using 1 μl of DNA template in a total reaction volume of 20 μl containing 10 μl of reaction buffer A, one μl of each upstream primer, downstream primer and probe (10 μmol/L), two μl of buffer B (containing 350 mmol/L magnesium acetate solution), and 5 μl of sterilized purified water. Thoroughly mix the reaction system and short-spin after preparation. A LightCycler 480II (Roche Diagnostics, USA) was applied for fluorescence signal collection every 30 s for 30 min at 39°C. Each test run contained negative and positive controls.

**Table 2:** The nucleic acid sequence of primers and probes tested in this study.

Types	Name	Nucleic acid sequences (5′-3′)
Upstream primer	F1	CATCCTGGGCATGTTCAACATGTTCTCGGG
	F2	CCCGTGCCGGGCATCGATCCGGATCAACTG
	F3	CATCGATCCGGATCAACTGGCKAAGCTGTTC
	F4	TCCTGGGCATGTTCAACATGTTCTCGGGYGGC
	F5	TCGCCGCAGCTCGARGCGCTGAAGAAGGAAGG
	F6	CACGATCTTYGCGCTGGGGATCATGCCGTAC
Downstream primer	R4	CCAGCGCRGCCGCGATACCGAAYGCCTGGAAG
	R5	CCCGGCTGGTTTTCCAGCGCRGCCGCGATAC
	R6	CACGACCGTCGTCAGHCGGAACAGCATGCCG
Probe	Probe	CAGGGCAACGGAAGATCACGCAGTACACGCGG [FAM-dT]A[THF][BHQ1-dT]TCACCGTGGTGCTCG-[C3Spacer]^a^

a FAM-dT: thymidine nucleotide carrying fluorescein; THF: tetrahydrofuran spacer; BHQ1-dT: thymidine nucleotide carrying Black hole quencher 1; C3Spacer: an oligonucleotide inhibitor structure.

### 2.4. *In silico* validations

The following *in silico* validations were conducted by Hangzhou Digital-Micro Co., Ltd. The whole genome database of bacteria was obtained from the NCBI website. The method of genome sequence quality control using CheckM (v1.0.18) was described elsewhere (Liang et al., 2021). Low-quality genomes with more than 5% of contamination or less than 90% of completeness or misnamed strains were removed from the database. After data cleaning, the genomic database contains 1,376 BCC strains and 156,793 non-BCC strains. Once the specific protein sequence was confirmed through the framework, the protein-coding gene and its conserved regions were delivered to the *in silico* validation process. The true-positive rate (sensitivity) and true-negative rate (specificity; Daddy Gaoh et al., 2021) of the gene and its core sequences were automatically analyzed using Blastn (version 2.13.0) to validate the presence of homologous gene sequences in the database. The Blastn parameters were set as follows: Identity>80, Query coverage>80%, and cut-off E-value <1e-5.

The estimating maximum-likelihood phylogenies of the specific gene sequences were constructed from 191 representative strains, including 47 strains of the known BCC species, 88 strains of the genus *Paraburkholderia* (Dobritsa and Samadpour, 2016), 27 strains of the genus *Caballeronia* (Uroz and Oger, 2017), 10 strains of the genus *Ralstonia* (Yabuuchi et al., 1995), and 19 strains of the genus *Burkholderia* strains. The GTR + R model of the IQtree (version 2.0.3) was used for phylogenetic analysis (bootstrap = 1,000; Nguyen et al., 2015). Furthermore, iTOL v5 was used to visualize the phylogenies (Letunic and Bork, 2021).

Before testing the artificially contaminated products, the primer and probe combination selected from preliminary tests was also validated *in silico* for sensitivity and specificity using the MFEprimer 3.2.4 (Wang et al., 2019) and the EMBOSS 6.6.0.0 (Rice et al., 2000), respectively. The binconf function from the Hmisc package (version 4.7–1) of R language is adopted, and Wilson confidence intervals are chosen for the confidence interval of *in silico* validation.

### 2.5. Experimental evaluations of the real-time RPA assay

#### 2.5.1. Inclusivity, exclusivity, and sensitivity study

The most appropriate primer and probe combination for the real-time RPA assay was further evaluated using the genomic DNA of the strains listed in Table 1 for the inclusivity and exclusivity study. Moreover, 10-fold serial dilutions of genomic DNA and bacteria in pure culture of *B. cepacia* CICC 10857 were tested to evaluate the assay’s sensitivity. The number of organisms in pure culture was determined by the colony counts method on SCDA, cultured overnight at 36°C ± 1°C. The concentrations of bacteria in pure culture were from 5.6 × 10^8^ CFU/ml to 5.6 × 10^2^ CFU/ml, respectively. The concentrations of genomic DNA were from 1.2 × 10^5^ pg/μl to 1.2 × 10^−2^ pg/μl, respectively.

#### 2.5.2. Artificial contamination study

Twelve pharmaceutical and 21 personal care products were sampled and inoculated with suitable concentrations of *B. cepacia* CICC 10857 for artificial contamination study. For the preparation of the samples, 10 g (for solid and semi-solid samples), 10 ml (for liquid samples), or 100 cm^2^ (for paste samples) were taken and mixed thoroughly with 90 ml of pH 7.0 sodium chloride peptone buffer to make a 1:10 test solution. Three replications were prepared for each sample. The inoculum amount of each replication was about 10 CFU, 1 CFU, or less than 1 CFU of the fresh bacterial suspension, respectively.

The pharmaceutical products were tested using the United States Pharmacopoeia General Chapter 〈60〉 as the reference method. Ten ml of the 1:10 test solution was added to 90 ml of TSB (Merck, USA). Then mix and incubate at 30–35°C for 24 h. The personal care products were tested using SN/T 4684–2016 standard in China, Determination of *Burkholderia cepacia* in cosmetics for import and export, as the reference method. Ten ml of the 1:10 test solution was added to 90 ml of Soybean casein digest lecithin polysorbate broth with 0.25 U/μl of polymyxin B (HuanKai Microbial, China). The cultures above were streaked on *Burkholderia cepacia* selective agar (BCSA, HuanKai Microbial, China) and incubated at 30–35°C for 48 h for the following confirmations required by the reference methods.

Meanwhile, 1 ml of each broth after incubation was centrifuged at 10,000 × *g* for 1 min. The precipitate was re-suspended in 500 μl of saline, boiled at 100°C for 10 min, and then cooled at room temperature. The supernatant containing genomic DNA was used directly in the real-time RPA assay. The results of the real-time RPA assay were compared with those of the reference methods, and the relative trueness and the relative level of detection of the real-time RPA assay were validated according to the sensitivity study of ISO 16140-2:2016.

## 3. Results

### 3.1. BCC-specific protein screening

After two screening steps, the BCC-specific biomarker mining procedure outputs various results using different screening parameters. When the parameter Identify was set as 90, 92, and 94 in the second step, there were 70 proteins, 8 proteins, and 2 proteins (LysR and SecY) output by the pipeline, respectively (detailed information was listed in Supplement data 6). Furthermore, there was one protein sequence that met the rigorous criteria (E-value = 1 × e^−10^, and Identify = 98) in the second step. This protein is identified as preprotein translocase subunit SecY (representative GenBank accession ID is AMU05241.1) according to the online BLASTp function of the NCBI website. It contains 449 amino acids and functionally relates to the translocation channel of the bacterial cell membrane (Flower, 2007; Dalal and Duong, 2011; Ma et al., 2019). The protein has 437 conserved amino acids within BCC bacteria by BLASTp analysis. All BCC species deposited in the GenBank database contained the associated protein sequence (Table 3). The core sequence of the *secY* gene is 268 bp in length, located at the site from 211 to 478 bp.

**Table 3:** SecY protein of BCC species deposited in the GenBank database.

No.	BCC species	GenBank accession ID for SecY protein
1	*B. cepacia*	ALK17286.1
2	*B. cenocepacia*	AMU05241.1
3	*B. contaminans*	BBA40776.1
4	*B. dolosa*	ETP66678.1
5	*B. multivorans*	QET39278.1
6	*B. pyrrocinia*	TDA45099.1
7	*B. stabilis*	VBB12013.1
8	*B. ubonensis*	KWN28506.1
9	*B. vietnamiensis*	ABO53363.1
10	*B. ambifaria*	ABI85847.1
11	*B. anthina*	KWZ34075.1
12	*B. arboris*	VWC30904.1
13	*B. diffusa*	AOI56359.1
14	*B. lata*	KML37261.1
15	*B. latens*	KVA09585.1
16	*B. metallica*	AOJ30269.1
17	*B. pseudomultivorans*	AOI90962.1
18	*B. seminalis*	AOJ23611.1
19	*B. stagnalis*	AOK51569.1
20	*B. territorii*	TXG27512.1
21	*B. aenigmatica*	UKD11786.1
22	*B. puraquae*	ORT81162.1
23	*B. catarinensis*	KAG8148618.1
24	*B. paludis*	KFG95783.1

**Figure 2:**
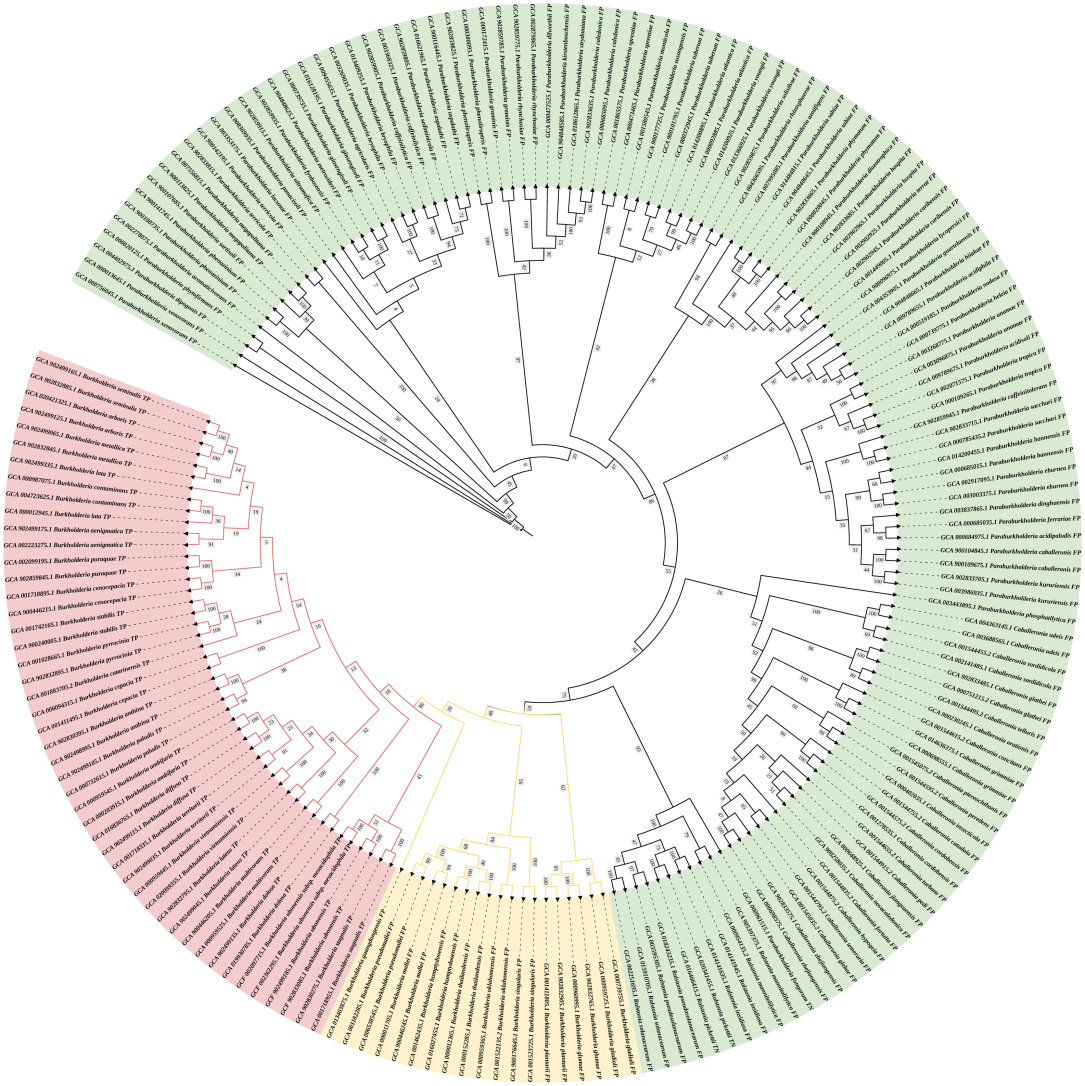
Phylogenetic tree of the *secY* gene in BCC bacteria and other closely related non-BCC bacteria (estimating maximum-likelihood method). The red branch refers to the BCC species, the yellow branch refers to the non-BCC species of the genus *Burkholderia*, and the green branch refers to other closely related species.

The DNA sequences of the protein-coding gene *secY* were extracted from the genomic database. The phylogenetic tree was constructed using 24 species of BCC bacteria, 10 non-BCC species of the genus *Burkholderia*, and other closely related species. The phylogenetic tree shows that all BCC species are clustered within one branch (red branch in Figure 2) and effectively distinguished from those non-BCC species. Therefore, the *secY* gene is a potential candidate for rapid BCC bacteria screening.

### 3.2. Preliminarily primer pairs evaluation

**Figure 3:**
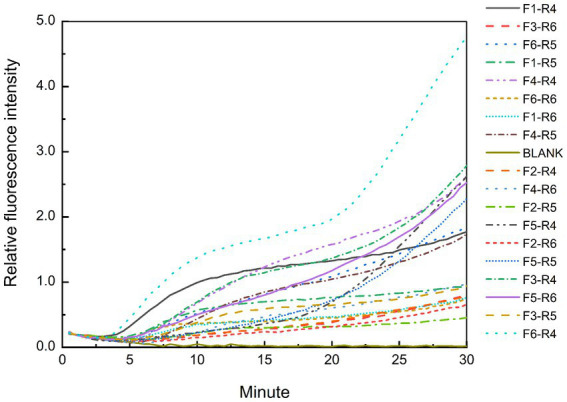
Test results of the real-time RPA assay for the genomic DNA of *B. cepacia* CICC 10857 using different primers and probe combinations.

The predicted sizes of the PRA amplicons are shown in Table 4. The genomic DNA of *B. cepacia* CICC 10857 was used to preliminarily evaluate the different combinations of primers and probes of the real-time RPA assay (Figure 3). The primer pairs of F1-R4, F3-R4, F4-R4, and F6-R4 showed better strength of fluorescence signals and fluorescence amplification curves. The combination that generated amplicons between 100 bp and 200 bp was considered a better RPA assay design (Lobato and O'Sullivan, 2018). Therefore, the F6-R4 primer pair with the assigned probe was confirmed for subsequent validation *in silico*.

**Table 4:** The predicted sizes of RPA amplicons by different primer combinations.

Amplicons/bp		Upper-stream primers					
		F1	F2	F3	F4	F5	F6
Down-stream primers	R4	241	300	289	239	123	193
	R5	254	313	302	252	136	206
	R6	300	359	348	298	182	252

### 3.3. *In silico* validations

The *in silico* evaluation of the *secY* gene was conducted for its full-length and core sequences with the genomic database containing 1,376 BCC and 156,793 non-BCC bacterial genomes (Table 5). The *secY* gene presented in nearly all BCC bacterial genomes, and the sensitivity was 99.93%. The specificity of the full-length sequence was 98.5% in our database. The only BCC strain missing the *secY* gene was *B. cenocepacia* VC2387 (GCA_001991115.1) in our database, presumably caused by incomplete sequencing data. Nevertheless, all other 336 genomes of *B. cenocepacia* strains showed the existence of the *secY* gene (strains listed in Supplement data 1). When validating the core sequence, the recognition of non-BCC strains (false positive results) sharply declined from 2,355 strains to 269 strains, while its sensitivity (99.27%) and specificity (99.8%) were not significantly affected. Therefore, the *secY* gene and its core sequence were proved to be a potential biomarker for rapid screening of BCC bacteria.

**Table 5:** Summary of the *in silico* validation for the *secY* gene.

Target sequences		Number of genomes		Sensitivity % (95% CL)^d^	Specificity % (95% CL)
		BCC	Non-BCC		
Full-length sequence	Positive^a^	1375 (TP)^b^	2,355 (FP)	99.93 (99.59–100.0)	98.50 (98.44–98.56)
	Negative	1 (FN)	154,438 (TN)		
Core sequence	Positive	1,366 (TP)	269 (FP)	99.27 (98.67–99.60)	99.80 (99.81–99.85)
	Negative	10 (FN)	156,524 (TN)		
F6-R4 primers	Positive	1095 (TP)^c^	7,388 (FP)	96.99 (95.82–97.84)	88.06 (87.80–88.31)
	Negative	34 (FN)	54,470 (TN)		
The primers and probe with ≤ 5 mismatches	Positive	1,095(TP)	0 (FP)	96.99 (95.82–97.84)	100.00 (99.99–100.00)
	Negative	34 (FN)	61,858 (TN)		

a Positive: the targets present in the genome. Negative: the targets do not present in the genome.

b TP: true-positive result; FN: false-negative result; FP: false-positive result; TN: true-negative result. Sensitivity = TP/(TP + FN); Specificity = TN/(FP + TN).

c The *in silico* validation of the primer and probe combination was performed using the representative bacterial genomes containing 1,129 BCC and 61,858 non-BCC bacterial genomes.

d Confidence Interval.

The F6-R4 primer combination was validated by 1,129 representative BCC and 61,858 non-BCC bacterial genomes (Table 5). The selected bacterial genomes are listed in Supplement data 2 and 3. The sensitivity of the F6-R4 combination was 96.99%, and 34 BCC strains belonging to 4 BCC species showed false negative results (Supplement data 4). Moreover, most false negative BCC species, such as *B. cepacia*, *B. cenocepacia*, *B. vietnamiensis*, and *B. ambifaria,* were subsequently demonstrated positive using the real-time RPA assay (Table 1). In addition, none of the selected representative non-BCC bacterial genomes showed any false positive results in the validation with the recognition of the primers and probe combination, allowing no more than five mismatches. The F6-R4 primer combination was considered an excellent choice to detect all known BCC strains in the database with reasonable confidence in sensitivity and specificity.

### 3.4. Inclusiveness and exclusivity of the real-time RPA assay

The real-time RPA assay was performed on the genomic DNA of 34 BCC strains and 24 non-BCC strains using the F6-R4 primer combination. All detection results were consistent with the identification and genetic information of the strains in Table 1. None of the false negative and false positive results were discovered. The real-time RPA assay could detect nine common BCC species (Genomovars I–IX) with 100% inclusiveness and exclusivity.

### 3.5. Sensitivity of the real-time RPA assay

**Figure 4:**
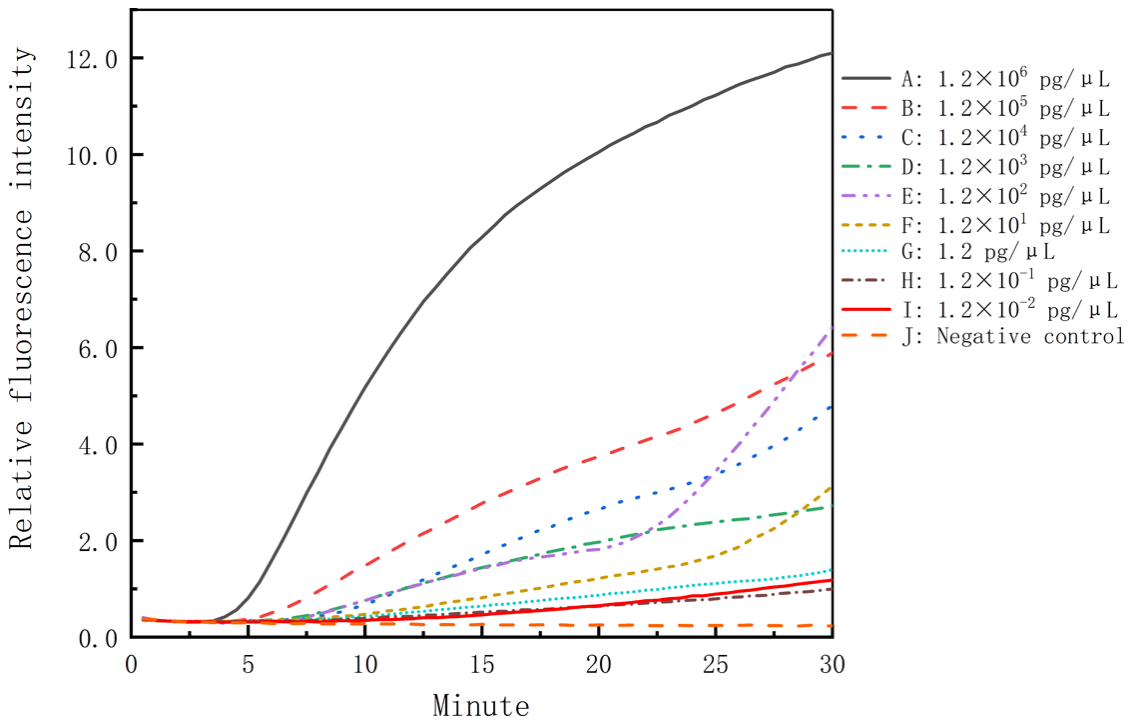
Sensitivity of the real-time RPA assay for the genomic DNA of *B. cepacia* CICC 10857. The symbols A-I in the figure represent the genomic DNA concentration of *B. cepacia* CICC 10857 as below, 1.2 × 10^6^ pg/μl, 1.2 × 10^5^ pg/μl, 1.2 × 10^4^ pg/μl, 1.2 × 10^3^ pg/μl, 1.2 × 10^2^ pg/μl, 1.2 × 10^1^ pg/μl, 1.2 pg/μl, 1.2 × 10^−1^ pg/μl, and 1.2 × 10^−2^ pg/μl, respectively. J: negative control.

The genomic DNA of *B. cepacia* CICC 10857 was serially diluted into 10-fold dilutions at concentrations from 1.2 × 10^6^ pg/μl to 1.2 × 10^−2^ pg/μl. These dilutions were used to validate the detection sensitivity of the real-time RPA assay. The sensitivity of the method was less than 1.2 pg/μl for BCC genomic DNA in 30 min with the F6-R4 combination (Figure 4).

We collected the genome information of 2,570 BCC strains deposited in Genbank from Oct 2005 to July 2023 (see Supplement data 5). The average size of BCC genomes is about 7.5 Mbp. Based on the calculation, the sensitivity of the *secY* gene was less than 1.46Χ10^2^ copies/μl by rough estimation.

**Figure 5:**
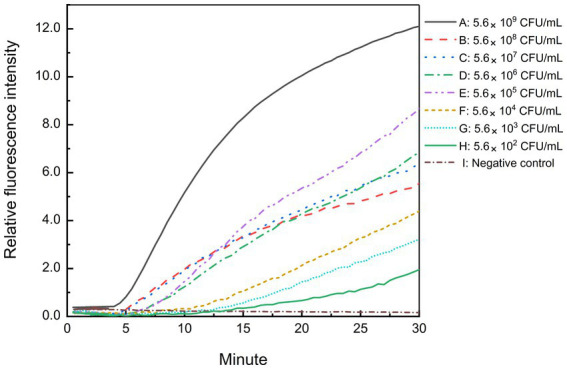
Sensitivity of the real-time RPA assay for *B. cepacia* CICC 10857 in pure culture. The symbols A-H in the figure represent the concentration of *B. cepacia* CICC 10857 in pure culture as below, 5.6 × 10^9^ CFU/ml, 5.6 × 10^8^ CFU/ml, 5.6 × 10^7^ CFU/ml, 5.6 × 10^6^ CFU/ml, 5.6 × 10^5^ CFU/ml, 5.6 × 10^4^ CFU/ml, 5.6 × 10^3^ CFU/ml, and 5.6 × 10^2^ CFU/ml, respectively. I: negative control.

Freshly cultured *B. cepacia* CICC 10857 was serially diluted into 10-fold serial dilutions at concentrations from 5.6 × 10^8^ CFU/ml to 5.6 × 10^2^ CFU/ml. The sensitivity of the real-time RPA assay for bacteria in pure culture was less than 5.6 × 10^2^ CFU/ml in 30 min with the F6-R4 combination (Figure 5). Pre-incubating of sample preparation in a broth medium is helpful and unavoidable to increase the detection sensitivity in actual samples before the molecular screening.

### 3.6. Validation of the artificially contaminated samples

The reference methods and the real-time RPA assay were performed and compared using the same test portion to carry out a paired study on 33 artificially contaminated samples inoculated in different bacterial concentrations. One hundred thirty-two pharmaceutical and personal care product test portions were analyzed (Table 6).

**Table 6:** The testing results of artificially contaminated samples using the reference methods and the real-time RPA method.

Categories	Samples	Inoculations and testing results							
		Sample without inoculation		≈0.1 CFU/portion		≈1 CFU/portion		≈10 CFU/portion	
		RM^a^	RPA	RM	RPA	RM	RPA	RM	RPA
Pharmaceutical products	Centella Triterpenes Cream	-^b^	−	+	+	+	+	+	+
	Compound Schisandra Syrup	−	−	−	−	+	+	+	+
	Guanjie Zhentong Babugao	−	−	−	−	+	+	+	+
	Dextromethorphan Hydrobromide and Guaifenesin Syrup	−	−	+	+	+	+	+	+
	Compound Antler Mixture	−	−	+	+	+	+	+	+
	Sodium Bicarbonate Ear Drops	−	−	+	+	+	+	+	+
	Lamivudine Oral Solution	−	−	−	−	+	+	+	+
	Paclitaxel Oral Solution	−	−	−	−	+	+	+	+
	Nucleotide and Casein Oral Solution	−	−	−	−	+	+	+	+
	Tiotropium Bromide Spray	−	−	−	−	+	+	+	+
	Ibuprofen Suspension	−	−	−	−	+	+	+	+
	Ephedrine Hydrochloride and Nitrofurazone Nasal Drops	−	−	+	+	+	+	+	+
Number of positive results		0	0	5	5	12	12	12	12
Personal care products	Mouthwash	−	−	−	−	+	+	+	+
	moisturizing cream	−	−	−	−	+	+	+	+
	Hyaluronic Acid Original Fluid	−	−	−	−	−	−	+	+
	Nourishing Balm	−	−	−	−	−	−	+	+
	Hydrolat floral water	−	−	−	−	+	+	+	+
	Eye cream	−	−	−	−	+	+	+	+
	Sunscreen	−	−	−	−	+	+	+	+
	Moisturizer	−	−	−	−	+	+	+	+
	Facial cleanser	−	−	−	−	+	+	+	+
	Moisturizing Refreshing Lotion	−	−	−	−	+	+	+	+
	Facial mask	−	−	+	+	+	+	+	+
	Shampoo	−	−	+	+	+	+	+	+
	Hair conditioner	−	−	+	+	+	+	+	+
	Body wash	−	−	−	−	+	+	+	+
	Makeup Remover	−	−	−	−	+	+	+	+
	Diaper rash Cream	−	−	−	−	+	+	+	+
	Facial treatment essence	−	−	−	−	+	+	+	+
	Baby moisture cream	−	−	−	−	+	+	+	+
	Baby moisture lotion	−	−	−	−	+	+	+	+
	Eyeshadow	−	−	−	−	+	+	+	+
	Lipstick	−	−	−	−	+	+	+	+
Number of positive results		0	0	3	3	19	19	21	21

a RM: Reference method. For pharmaceutical products, United States Pharmacopoeia General Chapter 〈60〉 was used as the reference method; and for personal care products, SN/T 4684–2016 standard was used as the reference method; RPA: real-time RPA assay in this study.

b −: negative result, +: positive result.

All samples without inoculation were confirmed as BCC-free according to the reference methods. The testing results from the reference methods and the real-time RPA assay maintained consistency for each sample portion. Besides the negative results from the hyaluronic acid original fluid and nourishing balm at the inoculation levels of 1 CFU/portion, the other testing results at the inoculation level or above were positive. Among the samples at the inoculation level of approximately 0.1 CFU/portion, the fractional positive rate was 24.2% (8/33). Based on the data summarized in Table 7, the relative trueness (RT) and sensitivity of the real-time RPA assay were 100% according to the ISO 16140-2:2016 standard, respectively. The relative level of detection (RLOD) was 1.0 compared with the reference methods for pharmaceutical and personal care products. Furthermore, no false positive or false negative results were observed in this study.

**Table 7:** Summary of results obtained from the real-time RPA assay and the reference methods for pharmaceutical and personal care products.

		Reference methods		Relative trueness %	Sensitivity%
		Positive	Negative		
Real-time RPA method	Positive	72 (TP)^a^	0 (FP)	100	100
	Negative	0 (FN)	27 (TN)		

a TP: true positive; FN: false negative; FP: false positive; TN: true negative. Relative trueness = (TP + TN)/(TP + TN + FN + FP); sensitivity = (TP + FP)/(TP+ FN + FP).

## 4. Discussion

The taxonomy of the genus *Burkholderia* is extraordinarily versatile and diverse, and it contains more than 120 species classified and separated from the genus *Pseudomonas* (Yabuuchi et al., 1992; Coenye et al., 2001c; Scoffone and Trespidi, 2021). BCC bacteria are genetically distinct but phenotypically similar bacteria within the genus *Burkholderia* (Yabuuchi et al., 1992; Mahenthiralingam et al., 2005). Because of the multi-drug resistance and a remarkable ability to survive in solutions (Geftic et al., 1979), BCC bacteria are at significant risk of infection in susceptible or immunosuppressed populations (Manu-Tawiah et al., 2001). BCC strains are frequently isolated in water-based products, especially in oral liquid solutions, nasal sprays, purified water, baby wipes, lotions, hand soaps, and mouthwashes (Kutty et al., 2007; Sutton and Jimenez, 2012; Marquez et al., 2017; Shaban et al., 2017; Solaimalai et al., 2019). Therefore, National drug regulatory authorities worldwide require manufacturers to monitor those objective microorganisms during production for microbiological risk assessment. However, due to the minor biochemical phenotypic differences, culture-based methods may give problematic detection and identification results with neither sensitivity nor specificity (Devanga Ragupathi and Veeraraghavan, 2019).

Molecular-based screening techniques present multiple advantages in rapid pathogen detection, such as rapidness, accuracy, and high sensitivity. It is a notable and influential improvement to the traditional methods in national or international standards. A well-studied specific molecular target is fundamental for methodology design. Evidence from DNA analysis showed that the sequence similarity within BCC species varies from 30 to 60% (Vandamme et al., 1997; Coenye et al., 2001a,b). Finding an ideal molecular detection target to cover all identified BCC bacteria is time-consuming, laborious, and frustrating.

Those commonly known conserved housekeeping genes are generally helpful for differentiating BCC species using various PCR, sequencing, or multi-locus sequence typing schemes (Coenye and Vandamme, 2003; Baldwin et al., 2005; Mahenthiralingam et al., 2008). For example, the *recA* gene shows 94 to 95% similarity between the genomovars of BCC bacteria (Mahenthiralingam et al., 2000), and species-level resolutions are efficiently achieved through the *recA* gene analysis for some problematic BCC strains (Cesarini et al., 2009; Vanlaere et al., 2009). In similar, the *rpsU* gene (Frickmann et al., 2014), *fur* gene (Lynch and Dennis, 2008)*, opcL* gene (Plesa et al., 2004), and *hisA* gene (Papaleo et al., 2010) are well achieved and applied in the literature. However, those housekeeping genes are found through a long-term selection and knowledge accumulation of gene or protein function studies in the literature by biological experts, which may require more than half a century (Lederberg and Tatum, 1946; Smith, 2012). The specific sequences as biomarkers with cell active functions for daily practice remain limited.

The bioinformatics comparison allows us to virtually validate and assess the characterization and performance of assays on a broader scale and in an easier way, especially for comparing newly emerged or re-classified species for an instant. In our study, the genomic data of newly reported BCC species are also introduced, such as the novel species *B. aenigmatica* (Depoorter et al., 2020), *B. paludis* (Shion et al., 2016), and *B. catarinensis* (Bach et al., 2017). Our automatic screening and evaluation framework is robust to mine biomarkers related to a particular function for BCC bacteria with lower synonymous mutations. The preprotein translocase subunit, protein SecY, is essential in forming an entirely proteinaceous pathway through which secreted proteins pass during membrane transit (Joly and Wickner, 1993; Driessen et al., 1998). Although some closely related non-BCC species and other bacteria contain the *secY* gene in their genomes according to bio-information analysis, such as *B. mallei, B. gladioli, B. plantarii, B. pseudomallei, B. glumae, Paraburkholderia rhizosphaerae, Paraburkholderia humisilvae, Paraburkholderia hospital,* and *Paraburkholderia steynii*, evolutionary analysis shows that their *secY* gene sequences differ significantly from those of BCC strains. Therefore, it demonstrates that the *secY* gene specific to BCC bacteria can distinguish BCC bacteria at a complex level and can assist in identifying new species of BCC bacteria.

Several previous sensitive molecular detection methods are based on the principles of half-nest PCR (Attia et al., 2016), real-time PCR (Jimenez et al., 2018), and lateral flow PRA (Peng et al., 2019) for detecting one or more BCC species. The primers or probes used in any detection method are suffered from limited experimental tests, and none of them are validated by all known species of BCC bacteria (Campbell 3rd et al., 1995; Jimenez et al., 2018). Insufficient validation will mislead industries and consumers with an uncertain risk of false negative results in process monitoring and control. Therefore, continuously re-validating these developed DNA-based methods to keep them up-to-date with increasing and newly emerged biodiversity should be considered (Coenye et al., 2001c). Although some molecular methods have been introduced and combined with DNA sequencing for accurate identification, confirmation, and discrimination among BCC bacteria (Mahenthiralingam et al., 2000; Payne et al., 2005; Vonberg et al., 2006; Pimentel et al., 2007; Martinucci et al., 2016), they are unsuitable for primary screening in daily practice.

In this study, we developed a novel BCC-specific biomarker mining strategy for the scientific community to screen reliable targets for microorganisms in metadata. We also provided an isothermal real-time RPA assay targeting the *secY* gene to rapidly screen suspicious BCC strains. The method has high specificity, accuracy, sensitivity, and low requirements for instrumentation. It is suitable for the risk control of BCC bacteria contamination in pharmaceutical and personal care products.

## Data availability statement

The original contributions presented in the study are included in the article/Supplementary material, further inquiries can be directed to the corresponding author.

## Author contributions

YLF: Conceptualization, Data curation, Formal analysis, Investigation, Methodology, Resources, Validation, Visualization, Writing – original draft. SJW: Formal analysis, Validation, Writing – original draft. MS: Resources, Writing – original draft. LLZ: Methodology, Software, Writing – review & editing. CZL: Validation, Writing – review & editing. YY: Validation, Writing – review & editing. SJY: Resources, Writing – review & editing. MCY: Funding acquisition, Project administration, Resources, Supervision, Writing – review & editing. All authors contributed to the article and approved the submitted version.

## Funding

The author(s) declare financial support was received for the research, authorship, and/or publication of this article. This work was supported by the Science and Technology Commission of Shanghai Municipality (22142201600 and 20DZ2293600), the National Key R&D Program of China (no. 2018YFC1603901), the Standard Improvement Project of Chinese Pharmacopoeia Commission (2022Y21), and the Scientific Research Project of Zhejiang Medical Products Administration (2023016).

## Conflict of interest

The authors declare that the research was conducted in the absence of any commercial or financial relationships that could be construed as a potential conflict of interest.

## Publisher’s note

All claims expressed in this article are solely those of the authors and do not necessarily represent those of their affiliated organizations, or those of the publisher, the editors and the reviewers. Any product that may be evaluated in this article, or claim that may be made by its manufacturer, is not guaranteed or endorsed by the publisher.

## Footnotes

^1^ www.ncbi.nlm.nih.gov/GenBank

^2^ https://github.com/zhou995287902/BCC_biomarker

^3^ https://blast.ncbi.nlm.nih.gov/Blast.cgi
